# Theory-based mHealth targeting fathers and mothers to improve exclusive breastfeeding: a quasi-experimental study

**DOI:** 10.1186/s13006-022-00537-x

**Published:** 2023-01-06

**Authors:** Kidane Tadesse Gebremariam, Afework Mulugeta, Danielle Gallegos

**Affiliations:** 1 School of Exercise and Nutrition Sciences, Institute for Physical Activity and Nutrition (IPAN), Deakin University, Melbourne, Australia; 2 School of Exercise and Nutrition Sciences, Queensland University of Technology (QUT), Brisbane, Australia; 3 School of Public Health, College of Health Sciences, Mekelle University, Mekelle, Ethiopia; 4 Woolworths Centre for Childhood Nutrition Research, Faculty of Health, Queensland University of Technology (QUT), Brisbane, Australia

**Keywords:** Breastfeeding, Exclusive breastfeeding, EHealth, MHealth, Partner support

## Abstract

**Background:**

Exclusive breastfeeding remains sub-optimal in low-income countries contributing to infant mortality. Mobile health (mHealth) interventions, delivered through personal mobile phones, to improve exclusive breastfeeding have shown promise, but very few include fathers or have been applied in low-income countries. The aim of this study was to assess the effectiveness of a SMS-based breastfeeding intervention targeting fathers and mothers in improving exclusive breastfeeding at three months in a low-income country.

**Methods:**

A quasi-experimental study was carried out with couples in their last trimester of pregnancy, at health centers, Mekelle, Tigray. This study was conducted from September 2018 to March 2019. The SMS-based intervention delivered a total of 16 SMS text messages to two arms: mothers-and-fathers, and mothers-only with the third group acting as the control. The main outcome measure was exclusive breastfeeding at months one, two and three after birth.

**Result:**

There were no significant differences in exclusive breastfeeding at month one between the three, mothers-and-fathers (95.1%), mother-only (90.2%), and control group (85%). At month three 85% of babies were exclusively breastfed in the mothers-and-fathers compared to 60% in the control group (*p* = 0.01). At month three 80% of babies were exclusively breastfed in the mothers-only compared to 60% in the control group (*p* = 0.04). In the multivariate analysis, babies born to mothers in the mother-and-fathers group were almost five times more likely to be exclusively breastfeed at three months than babies born to mothers who received standard care [AOR: 4.88, 95% CI (1.35,17.63)].

**Conclusion:**

An mHealth intervention targeting fathers and mothers, and mothers increased the likelihood of babies being exclusively breastfed at three months. The risk of not exclusively breastfeeding in the control group increased over time. A low-cost SMS-based breastfeeding intervention targeting fathers and mothers showed potential to improve exclusive breastfeeding. Such mHealth interventions could be integrated into the antenatal and postnatal follow-up services provided by midwives.

**Trial registration:**

This trial was registered with the Australian New Zealand Clinical Trials Registry (ANZCTR) 12,618,001,481,268.

**Supplementary Information:**

The online version contains supplementary material available at 10.1186/s13006-022-00537-x.

## Background

The World Health Organization (WHO) recommends infants be exclusively breastfed, with only breastmilk for the first six months of life [1]. Globally only one-third of infants are exclusively breastfed for the first six months [2, 3], with slightly higher (37%) rates in low-middle income country (LMIC) contexts [2]. In LMIC evidence indicates mortality risk for both non-breastfed and partially breastfed infants is higher compared to exclusively breastfed infants at the age of three months [4]. In sub-Saharan Africa, exclusive breastfeeding to the recommended six months decreases the risk of diarrheal disease, one of the major contributors to infant mortality, by 50% [5]. In Ethiopia, infant mortality has decreased significantly but remains at 43 deaths per 1000 live births, in part due to sub-optimal infant feeding practices [6]. According to national data, only 69 and 57%, of babies were being exclusively breastfed (EBF) at three months and six months, respectively [7]. Data shows that between 27 and 43% of infants in Ethiopia are exposed to a range of foods and liquids other than breastmilk [7–9] in the first few months of their life. At this early age, fluids such as plain water, milk, and sugar dissolved in water or juice or foods such as butter, are provided in addition to breastmilk [10]. Reducing this early introduction of food and fluids and encouraging EBF is an opportunity to decrease infant mortality and improve short and long-term overall infant and child health.

Interventions to improve EBF have largely tended to target mothers and focus on modifiable factors such as intention to breastfeed, breastfeeding self-efficacy, attitudes, knowledge, and social support [11–14]. There are, however, indications that interventions that include fathers are showing greater improvements in optimal breastfeeding practices [15, 16]. Data from LMICs show that involvement of fathers in breastfeeding interventions can improve mothers’ breastfeeding self-efficacy, knowledge, attitudes, and improves breastfeeding outcomes [17]. According to a systematic review of non-mHealth breastfeeding interventions targeting fathers in LMIC, there is a need for the development and evaluation of breastfeeding interventions involving fathers in low-income countries [17].

eHealth technologies such as web based, Short-Message Service (SMS), E-learning, and smartphone apps are known to have a positive effect on improving breastfeeding attitudes and knowledge and the duration of EBF [18]. SMS based mHealth interventions in LMIC targeting only mothers have improved early initiation of breastfeeding and EBF compared to mothers receiving standard care [19, 20]. A study within a high-income country context documented that mHealth interventions including fathers improved breastfeeding knowledge, attitudes, self-efficacy, early initiation and exclusive breastfeeding [21]. Considering the large number of hard-to-reach populations (rural/low income) with potentially lower access to health services, the rapid increase in mobile phone subscription and mobile network coverage in low-income countries [22] means that mHealth could play an important role in providing health education to improve breastfeeding knowledge, attitude, and self-efficacy. There are no known mHealth interventions including fathers based in LMIC.

In Ethiopia, mothers have mostly benefited from professional face-to-face support during antenatal (ANC) and postnatal care (PNC) [23], however, the proportion of mothers receiving their full complement of ANC and PNC is low at 41 and 13% respectively [8]. With the unprecedented increase in mobile phone access in low-and middle-income countries, mHealth could provide an opportunity to increase access to in-time ANC and PNC [24]. The expansion of the mobile phone network in Ethiopia, (currently around 46 million subscribers), could be an opportunity to improve the deliverability of breastfeeding education to parents through their mobile phone as an addition to the current ANC and PNC services offered by healthcare providers [25]. Given the rise of mobile phones, the very early introduction of foods and fluids prior to three months and relatively low rates of EBF in Ethiopia, the aim of this study was to investigate the effectiveness of a novel mHealth intervention targeting both fathers and mothers to improve breastfeeding knowledge, attitudes, maternal self-efficacy and partner support in order to improve EBF at three months.

## Methods

### Trial design

A quasi-experimental study design with three arms was conducted. In the first arm mothers and fathers (Mother-Father Intervention - MFI) received breastfeeding education through SMS in addition to standard care; in the second arm only mothers received the breastfeeding education through SMS (Mother’s only intervention - MI) in addition to standard care; and the third arm was the control group (CG) where couples received only standard care.

### Setting and participants

This research took place in health centers located in Mekelle, Ethiopia. Mekelle has nine public health centers, a tertiary hospital, and three general hospitals. Three public health centers with the highest ANC attendance rates that were at distance from each other were purposively (to get the proposed sample size and to avoid contamination) selected for study participant recruitment. The health centers were randomly (by lottery) assigned to the three intervention arms. Couples receiving services in each health center were assigned to one arm to avoid data contamination. All pregnant mothers in their last trimester and who had one month to their estimated date to give birth in the selected health centers were approached by nurses. First contact was made with mothers and then if they agreed to participate, fathers/partners were contacted by telephone. Couples who did not each have a personal mobile phone; who were not able to read and understand Tigrigna (the official language); were not living together, or where there were issues with the pregnancy or potential issues with breastfeeding were excluded from the trial.

### Randomization

**Fig. 1:**
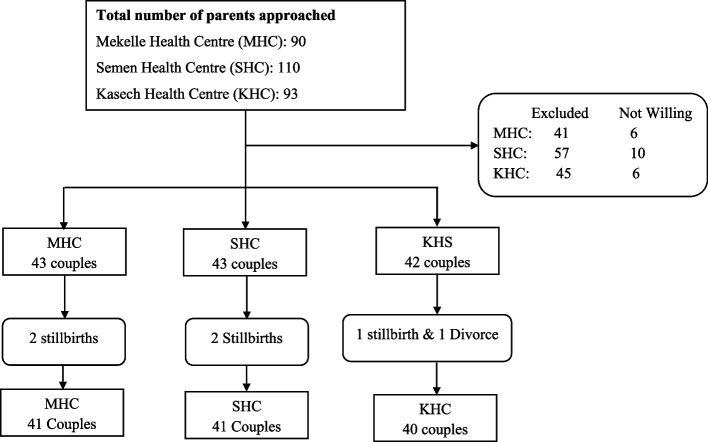
Flowchart of participants in the SMS based breastfeeding education intervention to improve exclusive breastfeeding in Mekelle, Tigray

Three community health centers were randomly assigned to the three arms. Couples who received pregnancy services in that health center were by default assigned to one of the arms of the intervention (Fig. 1).

### Theoretical framework

The theory of planned behavior (TPB) was used as the theoretical underpinning in the development of the SMS text message breastfeeding education approach. TPB is among many social science theories widely used to understand underlying health behaviors and for designing appropriate interventions and has been previously used in the development of breastfeeding interventions [12, 13]. According to the TPB, intention is the most proximal element of behavior. Behavioral intention is affected by attitudes related to the behavior, subjective norm (partner support), and perceived behavioral control (self-efficacy) [26]. The framework generally focuses on the cognitive or modifiable factors and can be used as a suitable theoretical context for designing interventions for behavioral change [26].

### Design of the SMS text messages

The content of the SMS breastfeeding education was developed after conducting an explorative qualitative study through focus groups discussion with fathers and mothers who had a child less than two years of age to inform the intervention. Based on the findings of the qualitative study [27] alignment with international breastfeeding recommendations, and the TPB, the research team developed 16 different weekly messages for fathers and mothers that aligned with prenatal and postnatal milestones and issues (Table 1). The intervention was delivered using a computer-based platform FrontlineSMS software program. The messages were automated “push” messages designed to reduce burden and cost for parents when considering interactive messaging in this setting.

**Table 1:** Antenatal and postnatal SMS text messages sent to fathers and mothers

Time	Fathers	Mothers
Antenatal SMS messages	Have you talked to your wife about breastfeeding your baby?	It may take a day or two for your milk to come in don’t give anything else but colostrum
	Breastmilk only will make your baby grow big, strong and smart	Always give your baby access to your breast so they can feed when they are hungry or thirsty
	The first milk colostrum is good for baby it will help the baby fight infection	The first milk colostrum is good for baby it will help the baby fight infection
	Ask the health workers to put your baby to your wife’s breast within an hour of giving birth	Let the baby suckle at your breast to increase milk supply
Postnatal SMS messages		
Month one	Breast milk has everything, and it is clean so that it helps brain development, to build the body, for health and its good for everything	When the baby has stomach pain bring the baby to the health facility, don’t give fenugreek
	Encourage your wife to breastfeed whenever the baby is hungry	Breastmilk is clean and safe and has enough water
	All your baby needs for the first six months is breastmilk, don’t give other food or liquid	Give your baby all of the breastmilk in one breast before starting on the other breast
	Feeding only breastmilk is important for your baby to grow big and strong	Ask your partner to bring you food and provide support
Month two	No water just breastmilk – water may give your baby diseases	Breastmilk has everything, and it is clean so that it helps brain development, to build the body, for health and its good for everything
	Help your wife, bring her a drink of water, soup or milk while she is breastfeeding	Breastmilk will protect your baby from diarrhoea
	Exclusive breastfeeding can protect from breast and cervical cancer	Exclusive breastfeeding can protect from breast and cervical cancer
	Tell the housemaid/grandmothers no water or food just breastmilk	All your baby needs for the first six months is breastmilk, don’t give other food or liquid
Month three	Help your wife express breastmilk into a cup if she is going out	Even if the baby is smelling don’t give food, wait until they are six months old
	Help your wife to breastfeed by doing the shopping	Don’t stop breastfeeding, you can overcome all challenges
	Encourage your wife to let the baby suckle at her breast to increase milk supply	Express breastmilk into a cup if you are going out
	Make sure your wife eats enough food – serve your wife if she is breastfeeding	Providing water, foods and liquids other than breastmilk will expose the baby to disease

### Message schedule

Couples included in the MFI each received a weekly breastfeeding SMS text message. Each parent (mother and father) in this group received four tailored SMS text messages during antenatal care (ANC) (for example “the first milk colostrum is good for baby it will help the baby fight infection”). After delivery, each parent received an additional twelve tailored postnatal (PNC) SMS text messages (for example “encourage your wife to let the baby suckle at her breast to increase milk supply”) through their personal mobile telephones. Each parent in this arm therefore, received a total of 16 weekly SMS text messages over a period of four months. Similarly, the mothers in the MI arm received the four ANC and twelve PNC, weekly SMS text messages through their personal mobile telephones. In addition to the breastfeeding intervention, couples in the MFI and MI received routine ANC and PNC care provided at their respective health centers. Couples in the control group received the ANC and PNC standard care provided at the health center they were attending. The delivery of all messages was tracked, and participants were asked separately about whether they read the messages, showed the messages to others and what they had learned. These data are not presented here.

### Study measurements

Where possible, all constructs were measured using tools validated across a range of international contexts, but not necessarily Ethiopia. A recognized process for cultural adaption of tools was used [28]. These tools were first translated into Tigrigna by the primary investigator. The translated questionnaires were then back translated by two public health nutritionists, with differences and comments discussed between the public health nutritionists and the research team. Some modifications were made in the Tigrigna version. Finally, face validity to ensure understanding and language was undertaken with ten mothers and ten fathers, after which some additional wording was changed.

The main outcome of the trial was the proportion of mothers who exclusively breastfed their babies at first, second, and third months. The definition of exclusive breastfeeding was based on the WHO indicator and included infants who were receiving breastmilk only. EBF was assessed using a 24-hour recall, one week recall, and one month recall [29] through phone interview with mothers. The inclusion of one week and one month ensured that any foods and fluids consumed since the last data point were also included.

The secondary outcomes of the intervention comprised breastfeeding attitudes, knowledge, and perceived partner breastfeeding support, for both mothers and fathers. These were collected through face-to-face interview at baseline (during the last trimester of pregnancy) and at the end of the study period (three months post-partum). Breastfeeding self-efficacy was measured for mothers only. Attitudes were measured using the Iowa Infant Feeding Attitude Scale (IIFAS) which has 17 questions and utilizes a Likert scale ranging from 1 = strong disagreement to 5 = strong agreement [30]. Total scores ranged from 17 to 85; with higher scores representing more positive attitudes. Partner breastfeeding support was assessed using the Partner Breastfeeding Influence Scale (PBIS) containing five sub-scales, breastfeeding savvy (learning about breastfeeding and discussing with partner), helping (providing tangible support), appreciation (encouraging and valuing breastfeeding partner), presence (partner’s assistance during breastfeeding), and responsiveness (father’s understanding to the mother’s needs). Each dimension was assessed using a five-point Likert scale from 1 (extremely not supportive) to 5 (extremely supportive). Mean scores were calculated for each breastfeeding support component [31]. A standardized tool from the Food and Agricultural Organization (FAO) of the United Nations (UN) [32] was used to assess breastfeeding knowledge. The tool has 10 open breastfeeding questions, which later coded to “Knows” or “Does not know”.

Maternal breastfeeding self-efficacy was measured using the Breastfeeding Self-efficacy Scale-Short Form (BSES-SF) [33]. The BSES-SF has 14 questions using a five-point Likert scale ranging from 1 (not at all confident) to 5 (always confident). Total scores range from 14 to 70. Higher scores indicated higher self-efficacy. In addition, the sociodemographic, economic, ANC service attendance, birth and infant, and infant characteristics were collected [11].

### Sample size

The sample size for the intervention was calculated using the power calculator and was estimated to be a total of 144 mothers and fathers that is 48 couples in each arm. The sample size was based on estimates of the proportion of EBF in the control group being 0.59 [34], and a 23% expected improvement in EBF in the experimental groups. The sample size was then calculated with the following assumptions α = 0.05, power = 80%, and an expected 10% attrition rate.

### Statistical analyses

Data were entered into IBM SPSS Statistics version 23 (IBM Corp. Released 2015. IBM SPSS Statistics for Windows, Version 23.0. Armonk, NY: IBM Corp). For categorical variables frequency with percent was reported while for continuous variables, either mean or median with standard deviation or interquartile range, respectively, were reported. Normality was tested for each continuous variable using variable inflation factor (VIF).

Chi square test was performed on the baseline characteristics of mothers and fathers. The differences in knowledge, attitude, and self-efficacy after the intervention between the three groups were determined with one-way ANOVA, or Kruskal Wallis test. Binary logistic regression was used to measure the effect of the intervention among the three groups. Baseline variables which were found to be significant (*p* < 0.05) in the chi square, or one-way ANOVA/Kruskal Wallis test were considered in the final logistic regression as confounders to test the effectiveness of the intervention. The level of significance was set at *p*-value < 0.05, where the null hypothesis was there was no intervention effect. The risk of stopping exclusive breastfeeding at month 1, month 2, and month 3 was expressed in terms of odds ratio with 95% CI.

## Results

At baseline, 43, 43 and 42 eligible couples were included in the mother and father intervention (MFI), mother-only intervention (MI) and control group (CG), respectively. There were no significant differences between the three groups at baseline for maternal age, maternal employment status, father’s age, father’s employment status, current child sex, and place of delivery. However, there were significant differences among the three groups in terms of fathers’ educational status, breastfeeding information during their ANC and fathers accompanying their partners to ANC (Table 2).

**Table 2:** Difference in participant characteristics across the three health centres Mekelle, Tigray

Variables	Mother-Father Intervention (*N* = 43) *n* (%)	Mother Intervention (*N* = 43) *n* (%)	Control Group (*N* = 42) *n* (%)	^a^*P*-value
Baseline mother age				
15-24 years	17 (39.5)	13 (30.2)	15 (35.7)	0.44
25-29 years	16 (37.2)	12 (27.9)	15 (35.7)	
30-39 years	10 (23.3)	18 (41.9)	12 (28.6)	
Educational status -mother				
Primary	14 (32.6)	12 (30.8)	13 (31.0)	0.08
Secondary	16 (37.2)	23 (59.0)	15 (35.7)	
Tertiary	13 (30.2)	4 (10.3)	14 (33.3)	
Employment -mother				
No job	17 (39.5)	23 (53.5)	20 (47.6)	0.13
Self-employed	12 (27.9)	16 (37.2)	13 (31.0)	
Employed	14 (32.6)	4 (9.3)	9 (21.4)	
Received BF information during ANC				
Yes	30 (69.8)	24 (55.8)	36 (85.7)	0.01*
No	13 (30.2)	19 (44.2)	6 (14.3)	
Breastfeeding Experience				
Yes	22 (51.2)	22 (51.2)	26 (61.9)	0.51
No	21 (48.8)	21 (48.8)	16 (38.1)	
Fathers’ age				
20-29 years	10 (23.2)	11 (25.6)	14 (33.4)	0.07
30-34 years	18 (41.9)	6 (14.0)	9 (21.4)	
35-39 years	9 (20.9)	12 (27.9)	10 (23.8)	
> =40	6 (14.0)	14 (32.5)	9 (21.4)	
Educational status - father				
Primary	22 (51.2)	9 (21.4)	11 (26.9)	0.01*
Secondary	15 (34.8)	16 (38.1)	16 (39.0)	
Tertiary	6 (14.0)	17 (40.5)	14 (34.1)	
Employment -father				
No job	3 (7.0)	6 (14.0)	5 (11.9)	0.19
Own job	18 (41.8)	25 (58.1)	24 (57.1)	
Employed	22 (51.2)	12 (27.9)	13 (31)	
Accompany your wife during ANC				
Yes	33 (76.7)	22 (51.2)	18 (42.9)	0.004*
No	10 (23.3)	21 (48.8)	24 (57.1)	
Child - Sex				
Male	19 (46.3)	16 (40)	20 (50)	0.66
Female	22 (53.7)	24 (60)	20 (50)	
Place of delivery				
Hospital	26 (63.4)	22 (53.7)	30 (75)	0.13
Health center	15 (36.6)	19 (46.3)	10 (25)	

*ANC* Antenatal Care, *BF* Breastfeeding

^a^*P*-value was based on chi-square test

*p < 0.05

At baseline there were significant differences between the three groups in mothers’ knowledge (*p* < 0.001), breastfeeding attitude (< 0.001), and in the perceived breastfeeding support, that is, breastfeeding savvy (*p* < 0.001), information (*p* < 0.001), appreciation (*p* < 0.001), presence (*p* < 0.001), and responsiveness (*p* < 0.001). There were no significant differences in fathers’ baseline breastfeeding, knowledge, savvy, help, appreciation, presence, or responsiveness. However, there were significant differences in fathers’ breastfeeding attitudes (0.02) between the three groups (Table 3).

**Table 3:** Baseline mean score differences in self-efficacy (only for mothers), knowledge, attitude, savvy, help, information, presence, and responsiveness of mothers and fathers in the three groups, Mekelle, Tigray

	Mothers				Fathers			
	Mother-Father Intervention (*N* = 43)	Mother only Intervention (*N* = 43)	Control Group (*N* = 42)		Mother-Father Intervention (*N* = 43)	Mother Intervention (*N* = 43)	Control Group (*N* = 42)	
Breastfeeding	Mean (+SD)	Mean (+SD)	Mean (+SD)	^a^*P*-value	Mean (+SD)	Mean (+SD)	Mean(+SD)	^a^*P*-value
Knowledge	65.7 (16.4)	67.6 (13.1)	60.2 (11.3)	< 0.001	60.9 (11.1)	64.6 (14.3)	57.3 (15.7)	0.06
Attitude	63.5 (7.4)	61.6 (6.4)	61 (8.3)	< 0.001	61.9 (9.2)	58.7 (7.1)	63.7 (8.5)	0.02
Savvy	36.6 (6.6)	30.6 (7.1)	41.9 (4.5)	< 0.001	38.9 (6.7)	36.6 (7.2)	38.8 (9.7)	0.34
Help	29.2 (4.7)	24.6 (4.4)	33.2 (3.2)	< 0.001	29.8 (6.2)	28.5 (5.4)	31.1 (6.5)	0.15
Information	25.7 (4.2)	21(4.6)	27.9 (3.4)	< 0.001	26.6 (4.2)	25.1 (4.3)	26.7 (6.4)	0.27
Presence	25.1 (4.1)	20.8 (4.5)	27.5 (3.5)	< 0.001	26.4 (4.4)	25 (3.9)	27.3 (5.4)	0.07
Responsiveness	20.4 (3.4)	17.5 (3.1)	22.2 (3.8)	< 0.001	21.1 (4.1)	20.6 (3.7)	22.1 (4.9)	0.30
Self-efficacy^b^	55.7 (6.1)	52 (9.8)	62 (6.9)	0.27				

^a^*P*-value was based on one-way ANOVA or Kruskal Wallis test

^b^Breastfeeding self-efficacy was measured only for mothers

### Breastfeeding practices

Attrition was less than 5 % and mainly due to neonatal death. As can be seen in Table 4, there were no significant differences in EBF at month one between the groups. However, at month two, 92.7% of babies born in the MFI group were exclusively breastfed, compared to 75% of the babies born in the CG (*p* = 0.04). At month three, 85.4 and 80.5% of the babies born in MFI, and MI groups were exclusively breastfed, compared to 60% of babies in the CG (*p* = 0.01, *p* = 0.04), respectively.

**Table 4:** Exclusive breastfeeding practices between intervention, and control groups at months one, two, and three in Mekelle, Tigray

	Mother-Father Intervention (*N* = 41)		MI: Mother only Intervention (*N* = 41)		Control Group (*N* = 40)
	*n* (%)	^a^*P*-value	*n* (%)	^a^*P*-value	*n* (%)
EBF Month-1					
Yes	39(95.1)		37(90.2)		34(85)
No	2(4.9)	0.14	4(9.8)	0.45	6(15)
EBF Month-2					
Yes	38(92.7)		35(85.4)		30(75)
No	3(7.3)	0.04*	6(14.6)	0.24	10(25)
EBF-Month-3					
Yes	35(85.4)		33(80.5)		24(60)
No	6(14.6)	0.01*	8(19.5)	0.04*	16(40)

Exclusive Breastfeeding: defined as infant receiving breastmilk only

^a^*P*-value was based on chi-square test

*P < 0.05

### Knowledge, attitudes, self-efficacy, and partner support at month three

There were significant mean differences in breastfeeding knowledge scores for mothers in the MFI, and MI intervention groups (Table 5). Similarly, mothers in the MFI group had more positive breastfeeding attitudes compared to mothers in the CG. With regard to breastfeeding support, mothers in the MFI group indicated that they received better support in terms of breastfeeding “savvy” and “information” from their partners compared to the MI and the CG (Table 5).

**Table 5:** Post intervention mean differences in knowledge, attitude, self-efficacy, savvy, help, information, presence, and responsiveness in the three groups Mekelle, Tigray

		Mothers		Fathers	
Breastfeeding variable	Group Comparisons	Mean Score Difference	^a^*P*-value	Mean Score Difference	^a^*P*-value
Knowledge	MFI-MI	0.98	0.91	8.04	0.004*
	MFI-CG	8.37	0.003*	4.3	0.19
	MI-CG	7.40	0.01*	−3.67	0.31
Attitude	MFI-MI	6.02	0.00*	6.04	0.001*
	MFI-CG	6.04	0.00*	9.13	0.001*
	MI-CG	0.02	1.00	3.08	0.03
Savvy	MFI-MI	4.17	0.001*	5.61	0.001*
	MFI-CG	3.20	0.01*	0.85	0.84
	MI-CG	−0.96	0.67	−4.75	0.006*
Help	MFI-MI	2.46	0.01*	2.68	0.01*
	MFI-CG	1.13	0.39	2.06	0.07
	MI-CG	−1.32	0.28	− 0.62	0.78
Information	MFI-MI	2.52	0.004*	2. 29	0.001*
	MFI-CG	2.37	0.008*	−0.01	1.00
	MI-CG	− 0.15	0.98	−2.30	0.001*
Presence	MFI-MI	1.20	0.26	−0.01	1.00
	MFI-CG	1.16	0.29	−0.79	0.66
	MI-CG	−0.04	0.99	−0.77	0.67
Responsiveness	MFI-MI	0.78	0.08	2.97	0.001*
	MFI-CG	0.22	0.82	0.26	0.88
	MI-CG	−0.57	0.27	2.71	0.001*
Self-efficacy	MFI-MI	2.61	0.19		
	FMI-CG	2.51	0.22		
	MI-CG	−0.10	0.99		

*MFI-MI* Mother-Father Intervention-Mother Intervention, *FMI-CG* Mother-Father Intervention-Control Group

*MI-CG* Mother Intervention-Control Group

^a^*P*-value was based on one-way ANOVA or Kruskal Wallis test

*P < 0.05

There was a significant mean difference in breastfeeding attitude score for fathers in the MFI compared to the fathers in the MI and the CG (*p* < 0.001). In addition, fathers’ support was significantly higher in the MFI, with fathers in the MFI tending to perceive they provided more breastfeeding support in the third month compared to the fathers in the MI and CG (Table 5).

According to the multivariable analysis, after controlling for potential confounding factors, including antenatal care attendance (Table 6), the breastfeeding intervention made a significant difference to breastfeeding exclusivity at month two and three, but there was no difference at month one. Babies who were born to mothers in the MFI were almost five times [AOR: 4.88, 95% CI (1.35,17.63)] more likely to be exclusively breastfed at month three compared to babies born to mothers in the control group. At month two, babies born to mothers in the MFI were six times more likely to be exclusively breastfed [AOR: 5.87, 95% CI (1.19,28.77)] compared to babies born to mothers in the control group (Table 6). Mothers with previous breastfeeding experience were found almost three times [AOR: 2.87, 95% CI (1.09,7.55)] more likely to exclusively breastfeed their babies at month three.

**Table 6:** Multivariable analysis showing the adjusted odds ratio at month-1, month-2, and month-3

Variable	Month-1: EBF AOR (95% CI)	Month-2: EBF AOR (95% CI)	Month-3: EBF AOR (95% CI)
Study group			
Mother-Father Intervention	3.11 (0.43, 22.20)	5.87* (1.19, 28.77)	4.88* (1.35, 17.63)
Mother only Intervention	1.56 (0.21, 11.61)	1.68 (0.36, 7.83)	2. 58 (0.68, 9.81)
Control Group	1.00	1.00	1.00
Previous breastfeeding experience			
Yes	3.13 (0.76, 12.91)	2.91 (0.94, 8.95)	2.87* (1.09, 7.55)
No	1	1.00	1.00
Breastfeeding information during ANC			
Yes	0.68 (0.12, 3.93)	0.57 (0.15, 2.13)	0.51 (0.17, 1.53)
No	1.00	1.00	1.00
Educational status of the father			
Primary	0.67 (0.12, 3.81)	0.65 (0.15, 2.71)	0.58 (0.17, 2.00)
Secondary	2.81 (0.45, 17.46)	0.88 (0.24, 3.16)	0.63 (0.21, 1.95)
Tertiary	1.00	1.00	1.00
Father accompanied during ANC			
Yes	1.86 (0.43, 8.11)	0.59 (0.19, 1.81)	0.69 (0.26, 1.80)
No	1.00	1.00	1.00
Baseline mothers’ breastfeeding knowledge	0.97(0.92, 1.03)	1.00 (0.96, 1.04)	0.99 (0.95, 1.03)
Baseline mothers’ breastfeeding self-efficacy	1.03 (0.96, 1.11)	0.99 (0.93, 1.06)	0.99 (0.94, 1.05)
Baseline fathers’ breastfeeding attitude	0.99 (0.90, 1.08)	0.98 (0.92, 1.05)	1.00 (0.95, 1.06)
Perceived partner’s breastfeeding responsiveness	81 (0.61, 1.08)	0.95 (0.82, 1.09)	1.02 (0.91, 1.13)

*ANC* Antenatal Care *p < 0.05

## Discussion

A SMS-based mHealth intervention involving fathers showed improvements in rates of EBF at three months. In addition, it improved mothers’ and fathers’ breastfeeding knowledge, attitudes, savviness, help, appreciation, and responsiveness at three months post-partum. Given that efforts to improve EBF in Ethiopia over the last five years, have showed an improvement of 1% in EBF (WHO definition) [6]; an mHealth intervention that focusses on both mothers and fathers could be a viable, potentially cost-effective option, to further improve optimal breastfeeding. This is the first known trial of an mHealth intervention for breastfeeding in a low resource setting that has focused on both mothers and fathers.

In the current study, participants in the SMS intervention were provided information regarding the importance of EBF, how to overcome breastfeeding challenges, and the role of fathers in breastfeeding. Although such information is expected to be provided during ANC and PNC follow-ups, many healthcare providers do not necessarily provide the required information to parents [8]. In addition, not all parents attend all PNC visits, and many do not attend together [35, 36]. While attendance at PNC was not collected, irrespective of the PNC provided, the provision of information through SMS text messaging to parents has contributed to improved EBF in the MFI group compared to the CG at two and three months and in the MI compared to CG at three months.

mHealth interventions in Africa targeting mothers have only increased EBF compared to mothers receiving standard care only [25, 26]. The involvement of fathers appears to have contributed to further improvements in EBF rates in the first three months. Previous mHealth interventions focusing on complementary feeding targeting fathers and mothers in Senegal have also shown significant improvements in infant and young child feeding (IYCF) behaviors [34]. Using SMS text messaging in breastfeeding interventions in a low resource setting could, therefore, improve EBF, and the involvement of fathers could further enhance these improvements.

A previous systematic review on mHealth interventions in LMICs indicated that the involvement of fathers in breastfeeding education improved EBF more than just mothers only [17]. According to Sahip & Turan, father’s involvement in breastfeeding education improved EBF by more than three times compared to the control group [37]. Previous assessments in Ethiopia have shown that although fathers indicated that they intended to support their partners during breastfeeding and childcare, they lacked knowledge, sources of information, and their partner’s perception about their involvement limited their involvement [23]. Ethiopian fathers have previously considered their support in terms of financial support to the family, leaving breastfeeding and childcare to the sole responsibility of the mother [23, 38].

Psychosocial factors, including maternal intention to breastfeed, breastfeeding self-efficacy, knowledge, and attitude affect the EBF practices of mothers [11, 39]. Interventions ranging from six weeks to six months designed based on the TPB have improved rates of EBF [12–14]. mHealth interventions targeting mothers in Taiwan and on mothers and fathers in Canada showed significant improvements in BF attitudes, knowledge and self-efficacy [21]. The current study also significantly improved BF knowledge and attitudes in mothers and fathers. However, there were no significant differences in maternal BF self-efficacy. A mHealth study, sending a text message a week, to mothers only in Australia also did not improve self-efficacy but did impact on “ways of coping” [20], indicating that changes to maternal self-efficacy may not be able to be detected when baseline self-efficacy is relatively high.

Controlling for other sociodemographic factors, mothers with previous breastfeeding experience were two times more likely to exclusively breastfeed their babies compared to first-time mothers [40, 41]. Similarly, the current study found that multiparous mothers were more likely to exclusively breastfeed their babies at month three compared to primiparous mothers. Future breastfeeding interventions should therefore consider breastfeeding experience during design to potentially develop different messages for mothers with different experiences.

This mHealth breastfeeding intervention targeting mothers and fathers has strengths and limitations, and some care should be taken with interpreting findings. First, this study is the first study mHealth intervention involving fathers in a low-income country. Secondly, the study incorporated messaging to cover both the antenatal and postnatal periods, many previously conducted breastfeeding interventions covered either antenatal or postnatal periods but not both [22]. Thirdly, the content of the mHealth intervention was developed through a co-design process with health experts and with fathers and mothers in the community. Finally, the study recruited 128 expectant couples and had a low attrition rate, and consequently was appropriately powered. This study has also limitations, due to time constraints the study could only follow couples one month antenatally and for the first three months after birth. It remains to be seen whether the intervention would have impacted on EBF at six months. This study was conducted with small sample size and significant differences between groups at baseline characteristics, thus, future interventions should consider larger sample size. The intervention was undertaken in an urban area with good mobile phone network coverage. Rural areas in low-income countries potentially have lower access to mHealth technologies and studies including urban and rural with a six-month follow-up is required to assess the feasibility and sustainability of such interventions. The tools used to measure the constructs underwent rigorous face validity process and in the main had been validated within an international context but did not undergo content validity in the Ethiopian context. Finally, data about parental participation in PNC at each health center were not captured, thus, future research should incorporate PNC service participation. Future studies should focus on extending the intervention to six months with larger sample size, to parents delivering in rural areas, to potentially engage grandmothers as key influencers of breastfeeding behavior and to undertake a cost-benefit analysis.

## Conclusion

A potentially low-cost SMS-based mHealth intervention that was co-designed with parents and health experts targeting mothers, and mothers and fathers increased exclusive breastfeeding rates at two and three months of age. The intervention also improved breastfeeding attitudes, knowledge and elements of perceived support. MHealth interventions to improve EBF in urban settings in a low-income country are feasible and involving fathers improves the effectiveness of the intervention. Such mHealth interventions could be integrated into the antenatal and postnatal follow-up services provided by midwives. This will help midwives and other staff in creating awareness among parents and will contribute to keeping them connected with health services antenatally and postnatally.

## Supplementary Information

**Additional file 1.**

**Additional file 2.**

## Data Availability

The datasets used for this study are available from the corresponding author on reasonable request.

## References

[1] World Health Organization. Indicators for assessing infant and young child feeding practices. Part I: Defintions1. Conclusions of a consensus meeting. Geneva: WHO; 2008.

[2] Victora CG, Bahl R, Barros AJD, França GVA, Horton S, Krasevec J (2016). Breastfeeding in the 21st century: epidemiology, mechanisms, and lifelong effect. Lancet.

[3] Xiaodong C, Tessa W, David WB (2012). Global trends in exclusive breastfeeding. Int Breastfeed J.

[4] Worl Health Organization (2016). Timing of initiation, patterns of breastfeeding, and infant survival: prospective analysis of pooled data from three randomised trials. Lancet Global Health.

[5] Ogbo FA, Agho K, Ogeleka P, Woolfenden S, Page A, Eastwood J (2017). Infant feeding practices and diarrhoea in sub-Saharan African countries with high diarrhoea mortality. PLoS One.

[6] Ethiopian Public Health Institute (EPHI) [Ethiopia], ICF (2019). Ethiopia mini demographic and health survey 2019: key indicators.

[7] CSA, ICF (2016). Ethiopia demographic and health survey 2016: key indicators report. Addis Ababa, Ethiopia, and Rockville.

[8] Central Statistical Agency Ethiopia. Ethiopia mini demographic and health survey 2014. Addis Ababa: Central Statistical Agency; 2014.

[9] Niguse T, Frehiwot H, Dinu A, Eyerus D (2016). Knowledge, attitude and practice towards exclusive breastfeeding among lactating mothers in Mizan Aman town, southwestern Ethiopia: descriptive cross-sectional study. Int Breastfeed J.

[10] Mulugeta A, Hagos F, Kruseman G, Linderhof V, Stoecker B, Abraha Z (2010). Factors contributing to child malnutrition in Tigray. Northern Ethiopia East Africa Medical Journal.

[11] Meedya S, Fahy K, Kable A (2010). Factors that positively influence breastfeeding duration to 6 months: a literature review. Women and Birth.

[12] Yu Z, Zhihong Z, Yun L, Hongwei W. Impact of intervention on breastfeeding outcomes and determinants based on theory of planned behavior. Women and Birth. 2017;30:146–52. 10.1016/j.wombi.2016.09.011 27773609

[13] Yanhong G, Yu Z, RN M, Zhihong Z, Hongwei W (2016). Effectiveness of a theory-based breastfeeding promotion intervention on exclusive breastfeeding in China: a randomised controlled trial. Midwifery..

[14] Guo JL, Wang TF, Liao JY, Huang CM (2016). Efficacy of the theory of planned behavior in predicting breastfeeding: Meta-analysis and structural equation modeling. Appl Nurs Res.

[15] Abbass-Dick J, Stern SB, Nelson LE, Watson W, Dennis C-L (2015). Coparenting breastfeeding support and exclusive breastfeeding: a randomized controlled trial. Pediatrics..

[16] Bar-Yam NB, Darby L (1997). Fathers and breastfeeding: a review of the literature. J Hum Lact.

[17] Tadesse K, Zelenko O, Mulugeta A, Gallegos D (2018). Effectiveness of breastfeeding interventions delivered to fathers in low- and middle-income countries: a systematic review. Maternal and Child Nutrution.

[18] Ying L, Htun TP, Tam WSW, Klainin-Yobas P (2016). Efficacy of e-technologies in improving breastfeeding outcomes among perinatal women: a meta-analysis. Maternal and Child Nutrition.

[19] Flax VL, Negerie M, Ibrahim AU, Leatherman S, Daza EJ, Bentley ME (2014). Integrating group counseling, cell phone messaging, and participant-generated songs and dramas into a microcredit program increases Nigerian women's adherence to international breastfeeding recommendations. J Nutr.

[20] Gallegos D, Russell-Bennett R, Previte J, Parkinson J (2014). Can a text message a week improve breastfeeding?. BMC Pregnancy and Childbirth.

[21] Abbass-Dick J, Xie F, Koroluk J, Alcock Brillinger S, Huizinga J, Newport A (2017). The development and piloting of an eHealth breastfeeding resource targeting fathers and partners as co-parents. Midwifery..

[22] Ericsson. Ericsson mobility report: on the pulse of the networked society. Sweden JUNE 2015. Report No.: 3.

[23] Alive & Thrive (2010). Practices, IYCF practices, beliefs, and influences in Tigray region, Ethiopia.

[24] Bastawrous A, Hennig B, Livingstone I (2013). mHealth possibilities in a changing world. Distribution of global cell phone subscriptions. J Manuf Technol Manag.

[25] World Bank. Mobile cellular subscriptions: World Bank 2016 [Available from: https://data.worldbank.org/indicator/IT.CEL.SETS.P2?end=2017&locations=ET&start=1960&view=chart.

[26] Ajzen I (1991). Theory of planned behavior. Organ Behav Hum Decis Process.

[27] Gebremariam KT, Zelenko O, Hadush Z, Mulugeta A, Gallegos D (2020). Exploring the challenges and opportunities towards optimal breastfeeding in Ethiopia: a formative qualitative study. Int Breastfeed J.

[28] Beaton D, Bombardier C, Guillemin F, Ferraz M (2001). Guidelines for the process of cross-cultural adaption of self-report measures. Spine..

[29] Greiner T (2014). Exclusive breastfeeding: measurement and indicators. Int Breastfeed J.

[30] Adl M, Russell DW, Dungy CI, Mary L, Lois D (1999). The Lowa infant feeding attitude scale: analysis of reliability and validity. J Appl Soc Psychol.

[31] Rempel LA, Rempel JK, Moore KCJ (2017). Relationships between types of father breastfeeding support and breastfeeding outcomes. Matern and Child Nutrition.

[32] Marías YF, Glasauer P. Guidelines for assessing nutrition-related knowledge, attitudes and practices. Rome: Food and Agriculture Organization of the United Nations (FAO). 2014:vi–+ 180.

[33] Dennis C-L (2003). The breastfeeding self-efficacy scale:psychometric assessment of the short form. J Obstet Gynecol Neonatal Nurs.

[34] Downs SM, Sackey J, Kalaj J, Smith S, Fanzo J (2019). An mHealth voice messaging intervention to improve infant and young child feeding practices in Senegal. Maternal and Child Nutrition.

[35] Tekelab T, Chojenta C, Smith R, Loxton D (2019). Factors affecting utilization of antenatal care in Ethiopia: a systematic review and meta-analysis. Plos one.

[36] Mohammed BH, Johnston JM, Vackova D, Hassen SM, Yi H (2019). The role of male partner in utilization of maternal health care services in Ethiopia: a community-based couple study. BMC Pregnancy and Childbirth..

[37] Sahip Y, Turan JM (2007). Education for expectant fathers in workplaces in Turkey. J Biol Sci.

[38] USAID/ENGINE. Fathers’ infant and young child feeding practices and their determinants in Amhara, Oromia, SNNP and Tigray Regions. Addis Ababa: USAID/ENGINE; 2014.

[39] de Jager E, Broadbent J, Fuller-Tyszkiewicz M, Skouteris H (2014). The role of psychosocial factors in exclusive breastfeeding to six months postpartum. Midwifery..

[40] Dachew BA, Berhanu BB (2014). Breastfeeding practice and associated factors among female nurses and midwives at North Gondar zone, Northwest Ethiopia: a crosssectional institution based study. Int Breastfeed J.

[41] Jessri M, Farmer AP, Maximova K, Willows ND, Bell RC (2013). Predictors of exclusive breastfeeding: observations from the Alberta pregnancy outcomes and nutrition (APrON) study. BMC Pediatr.

