# Supplementary material for: Theory-based mHealth targeting fathers and mothers to improve exclusive breastfeeding: a quasi-experimental study
Source: Int Breastfeed J. 2023 Jan 6;18:2. doi: 10.1186/s13006-022-00537-x (PMC9817286; doi:10.1186/s13006-022-00537-x)
Supplement: Supplementary file 1 — Additional file 1. [file 13006_2022_537_MOESM1_ESM.docx]

Ethical approval and consent to participate: This study obtained ethical approval from the Human Research Ethics Committee (HREC) of Queensland University of Technology (QUT), UHREC: 1700000717, Australia; and the Health Research Ethics Review Committee (HRERC) of Mekelle University, Ethiopia, ECR: 1194/2017. All participants provided written informed consent.

Consent for publication: Not applicable

Acknowledgements: We would gratefully like to thank the study participants, Tigray Regional Health Bureau, and directors of the health centers for facilitating the research, and the data collectors for their help in recruitment and conducting the data collection. We are grateful to Dr. Oksana Zelenko for her valuable support during the design of co-design process and significant

contribution throughout the implementation process.

Funding: This work was supported by Queensland University of Technology (QUT) PhD student allocation and the Child and Adolescent Health (CAH) theme of Institute of Health and Biomedical Innovation (IHBI), QUT. These funding institutions have not played a role in the design, analysis or preparation of this manuscript.

Conflict of Interest: Danielle Gallegos is currently Chair of the Woolworths Centre for Childhood Nutrition Research. This is funded by the Queensland Children’s Hospital Foundation through a philanthropic donation from Woolworths. Woolworths and the Queensland Children’s Hospital Foundation played no role in the conduct or reporting of this research.

Author contributions: KTG participated in design, data collection, analysis, and wrote the original manuscript. DG participated in design, analysis and manuscript revision. AM participated in design and manuscript revision.
