# Supplementary material for: Theory-based mHealth targeting fathers and mothers to improve exclusive breastfeeding: a quasi-experimental study
Source: Int Breastfeed J. 2023 Jan 6;18:2. doi: 10.1186/s13006-022-00537-x (PMC9817286; doi:10.1186/s13006-022-00537-x)
Supplement: Supplementary file 2 — Additional file 2. [file 13006_2022_537_MOESM2_ESM.docx]

Theory-based mHealth targeting fathers and mothers to improve exclusive breastfeeding: a quasi-experimental study

Kidane Tadesse Gebremariam BSc, MSc., PhD ^1, 2, 3*^ (gkidanet@gmail.com), Afework Mulugeta BSc., MSc., PhD^3^ ([afework.mulugeta@gmail.com](mailto:afework.mulugeta@gmail.com)), Danielle Gallegos BSc., Grad Dip Nut & Diet, PhD, FDAA^2,4^([danielle.gallegos@qut.edu.au](mailto:danielle.gallegos@qut.edu.au))

^1^ Deakin University, Geelong, Australia, Institute for Physical Activity and Nutrition (IPAN), School of Exercise and Nutrition Sciences

^2^ School of Exercise and Nutrition Sciences, Queensland University of Technology (QUT), Victoria Park Road Kelvin Grove, QLD, 4059, Australia

^3^ School of Public Health, College of Health Sciences, Mekelle University, Mekelle, Ethiopia

^4^ Woolworths Centre for Childhood Nutrition Research, Faculty of Health, Queensland University of Technology (QUT), Graham St, South Brisbane, QLD, 4101, Australia

*Corresponding author

Kidane Tadesse Gebremariam

[gkidanet@gmail.com](mailto:gkidanet@gmail.com)

Mobile; +61468618270

Word count: Abstract (300), and Text (3550)
